# Population Genetic Structure of the Magnificent Frigatebird *Fregata magnificens* (Aves, Suliformes) Breeding Colonies in the Western Atlantic Ocean

**DOI:** 10.1371/journal.pone.0149834

**Published:** 2016-02-22

**Authors:** Andressa Nuss, Caio J. Carlos, Ignacio B. Moreno, Nelson J. R. Fagundes

**Affiliations:** 1 Departament of Genetics, Institute of Biosciences, Federal University of Rio Grande do Sul, Porto Alegre, RS, Brazil; 2 Laboratório de Sistemática e Ecologia de Aves e Mamíferos Marinhos (LABSMAR), Departament of Zoology, Institute of Biosciences, Federal University of Rio Grande do Sul, Porto Alegre, RS, Brazil; University of Padova, ITALY

## Abstract

The Magnificent Frigatebird *Fregata magnificens* has a pantropical distribution, nesting on islands along the Atlantic and Pacific coasts. In the Caribbean, there is little genetic structure among colonies; however, the genetic structure among the colonies off Brazil and its relationship with those in the Caribbean are unknown. In this study, we used mtDNA and microsatellite markers to infer population structure and evolutionary history in a sample of *F*. *magnificens* individuals collected in Brazil, Grand Connétable (French Guyana), and Barbuda. Virtually all Brazilian individuals had the same mtDNA haplotype. There was no haplotype sharing between Brazil and the Caribbean, though Grand Connétable shared haplotypes with both regions. A Bayesian clustering analysis using microsatellite data found two genetic clusters: one associated with Barbuda and the other with the Brazilian populations. Grand Connétable was more similar to Barbuda but had ancestry from both clusters, corroborating its “intermediate” position. The Caribbean and Grand Connétable populations showed higher genetic diversity and effective population size compared to the Brazilian population. Overall, our results are in good agreement with an effect of marine winds in isolating the Brazilian meta-population.

## Introduction

The Magnificent Frigatebird *Fregata magnificens* is a large seabird measuring 89–114 cm long with a 217–244 cm wingspan that breeds on islands along the Pacific and Atlantic coasts of the Americas. In the Pacific, breeding colonies can be found from Baja California (Mexico) to Ecuador, including the Galapagos Islands; in the Atlantic, colonies occur from Florida and the Caribbean to south Brazil. There is also a very small population on the Cape Verde Islands off western Africa [1]. In Brazil, breeding colonies can be found on the Fernando de Noronha archipelago, which is located 350 km off the Brazilian coast in the Equatorial South Atlantic, and on other inshore archipelagos along the country’s eastern coast, between latitudes 17–27μS [2].

Similar to its congeners, *F*. *magnificens* is a powerful flyer and can glide for hours without flapping its wings [3]. Weimerskirch *et al*. [4] equipped some birds from Grand Connétable, off French Guiana, with altimeters and satellite transmitters to record their vertical and horizontal movements. Their results shown that Magnificent Frigatebirds spend days and nights soaring on the relatively weak oceanic thermals, covering distances of up to 200 km before landing [4]. Frigatebirds have the lowest wing-load (*i*.*e*., the ratio between body mass and wing area) of any bird species, allowing them to soar efficiently on weak oceanic thermals [1], [3], [4].

In view of its flight power, it would be expected that *F*. *magnificens* populations exhibit high gene flow across large geographic distances. However, seabird populations in general display genetic structure in spite of high dispersal ability [5], suggesting that other ecological factors may restrict gene flow. For example, philopatry and coloniality may be beneficial [6] and represent a survival strategy in marine environments [5]. As a result, gene flow in large marine vertebrates such as seabirds and cetaceans may be more dependent on wind and hydrographic characteristics, including the temperature, seasonality, salinity, and nutrient concentrations of water and air masses [6], [7].

A previous study with *F*. *magnificens* from several breeding colonies in the Pacific and central Atlantic Ocean indicated extensive gene flow among populations (except for the isolated Galapagos population) based on mitochondrial DNA (mtDNA) and microsatellite (STR) markers, and low mtDNA haplotype diversity compared to terrestrial birds [8]. However, because no breeding colonies off Brazil were included in this study, the genetic connection between the Caribbean and South Atlantic populations is unknown. From the conservationist point of view, *F*. *magnificens* is listed as of high concern in the North American Waterbird Conservation Plan [9], but as least concern in IUCN [10]. However, the IUCN justification ignore the potential effects of strong genetic structure among the different oceanic basins that may indicate the presence of different evolutionary groups in this species, each deserving special attention in conservation policies [11], [12]. Therefore, knowledge about population structure in this species across its entire distribution is fundamental for planning conservation strategies, and may help to understand evolutionary processes responsible for diversification in tropical oceanic fauna.

In this study, we sampled individuals from breeding colonies off South America and in the Caribbean to characterize the pattern and level of genetic structure in this species across different oceanic basins using mtDNA and a set of microsatellite (SSR) markers. More specifically, we asked the following questions: Is there significant genetic structure among colonies from the Atlantic Ocean, including populations from South Atlantic Ocean? Are levels of genetic diversity similar among colonies and oceanic basins? Do oceanic wind patterns provide a good explanation for the patterns revealed by the genetic markers?

## Material and Methods

### Ethics Statement

The study was carried out under the approval of the competent federal agency in all countries: Ministério do Meio Ambiente, Brazil—SISBIO 37193–2, given to AN for collecting samples in three populations. Permits number SISBIO 16553–1 and 23134–3 were given to researchers who sent tissue samples for us and are nominated in the Acknowledgments. The same situation applies for permits given by the Barbuda Council, Antigua and Barbuda National Parks, and the Environmental Awareness Group of Antigua, as well as the French Guiana’s Regional Environmental Authority—DIREN.

### Samples and Laboratory Analyses

**Fig 1:**
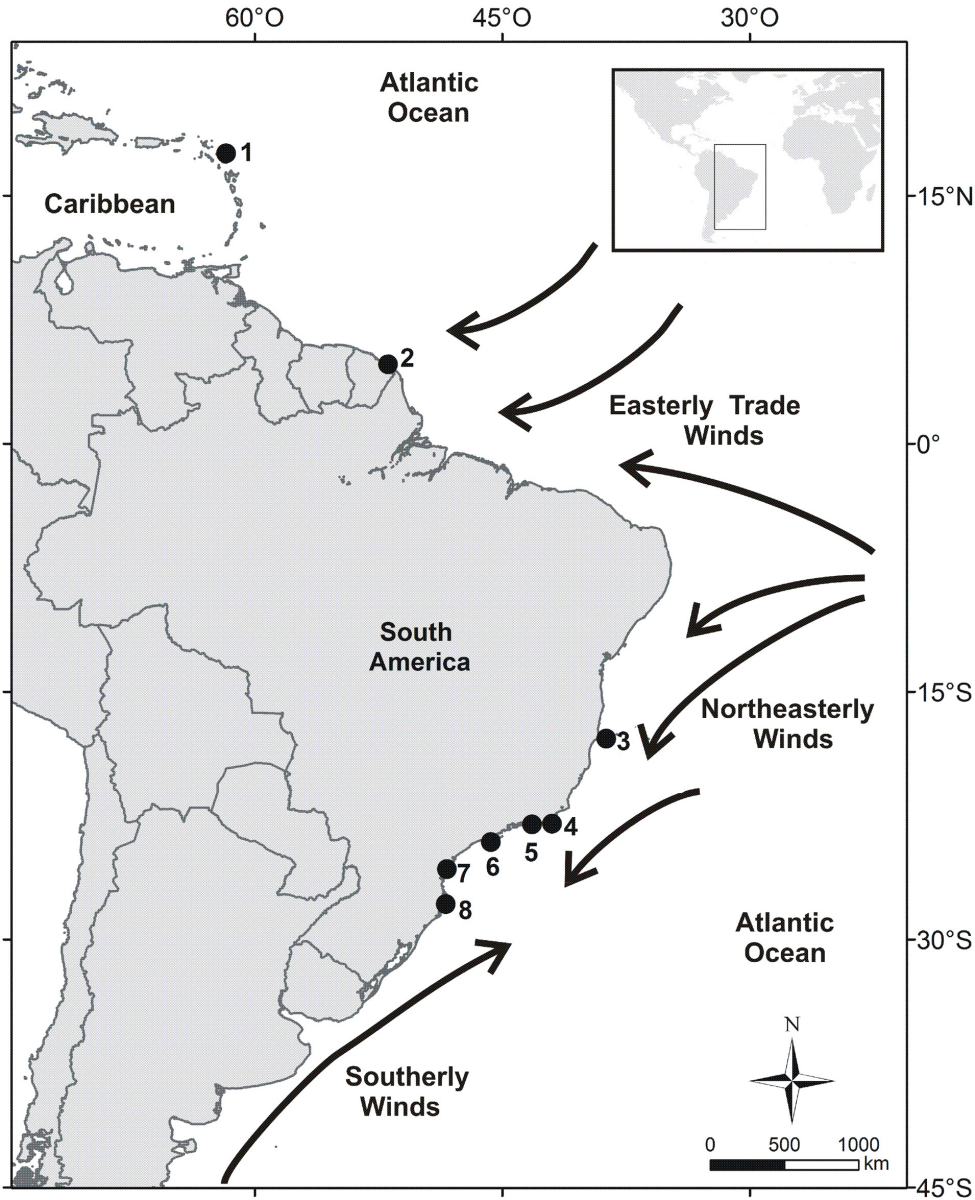
Map of sampled populations. 1- Barbuda; 2- Grand Connétable; 3- Abrolhos; 4- Cabo Frio; 5- Cagarras; 6- Alcatrazes; 7- Currais; 8- Moleques do Sul. Major global wind patterns discussed in the main text are also shown.

Overall, we collected samples from 156 individuals from eight populations in the Atlantic, including one population in the Caribbean (Barbuda [17μ35'N; 61μ45'W]), one in northern South America (Grand Connétable, French Guiana [4°49’N; 51°56’W]), and six along the eastern Brazilian coast (Abrolhos [17μ50'S; 38μ42'W], Cabo Frio [22μ59' S; 41μ59' W], Cagarras [23μ02'S; 43μ12'W], Alcatrazes [24μ06'S; 45μ42'W], Currais [25°44’S; 48°22’W], Moleques do Sul [27μ51’S; 48μ26’W]) (Fig 1). We sampled different tissues for DNA extraction: fresh tissue from embryos; muscles, paw scales, and nails from carcasses; blood collected via brachial venipuncture or using filter paper; and feather tips from both old and recently collected feathers. Chicks comprised 77% of our sample and were presumably unrelated at least concerning the current generation. Except for the filter paper and feathers, all other samples were stored in absolute ethanol.

DNA was extracted using the PureLink Genomic DNA Mini Kit (Invitrogen) or the Quick-gDNA MiniPrep (Zymo Research). We also extracted DNA following two standard protocols based on proteinase K digestion followed by salting-out (adapted from Medrano *et al*. [13]) or CTAB digestion and phenol:chloroform purification (adapted from Doyle & Doyle, [14]). We tested different methods for each type of sample, and some samples were extracted more than once with several protocols to obtain high-quality DNA. All samples were quantified using Picodrop and diluted to 40 ng/μl for use.

We amplified fragments of two mitochondrial genes, NADH dehydrogenase subunit 2 (ND2) and cytochrome b (cytb), using primers described previously ([8] and [15] respectively). We used cytb primers given in [15] instead of the species-specific sequences from [8] because these latest set of oligonucleotides failed to produce good PCR amplifications after several trials. There is evidence of mtDNA duplication in some seabird species that includes a partial copy of the cytb gene [16]. However, we amplified an amplicon larger than all known partial cytb copies and found some haplotypes identical to those reported by Hailer *et al*. [8] using species-specific primers, strongly suggesting that we are studying the functional cytb copy. The PCR conditions were the same for the two mitochondrial segments: 10 cycles of 94°C for 60 s, 60°C for 60 s (-1μC/cycle), and 72°C for 60 s, followed by 35 cycles of 94°C for 60 s, 55°C for 60 s, and 72°C for 60 s, and a final extension of 72°C for 10 min. The amplified products were assessed on a 1% agarose gel, purified enzymatically with shrimp alkaline phosphatase and exonuclease I (GE Healthcare), and Sanger sequenced by Macrogen Inc., South Korea.

We selected eight microsatellite (Short Tandem Repeat—STR) loci (Fmin02, Fmin11, Fmin12, Fmin14, Fmin15, Fmin16, Fmin17, and Fmin18; [17]) developed for the Great Frigatebird, *F*. *minor*, which were shown to exhibit consistent amplification and polymorphism in *F*. *magnificens* [8] Every forward primer was 5’-tailed with an M13 sequence (5’-CACGACGTTGTAAAACGAC-3’) and used in combination with an M13 primer that had the same sequence but was dye labelled at its 5’ end [18]. The PCR reactions were performed individually for each locus in a 10-μl volume containing 1 μl of diluted DNA, 1x PCR Buffer (Invitrogen), 2.5 mM MgCl_2_, 0.2 mM dNTPs, 0.4 μM of reverse and M13-fluorescent primers, 0.05 μM of M13-tailed forward primer, and 0.1 unit of Taq Platinum DNA Polymerase (Invitrogen). The cycling parameters were as follows: 94°C for 5 min; 10 touchdown cycles in which annealing temperature varied between 65°-60°; 35 cycles of 94°C for 45 s, 60°C for 45 s, and 72°C for 45 s; and one final cycle for 15 min at 72°C. Following amplification, the PCR products were diluted, and different loci were pooled together based on the expected size range and fluorescent dye. Genotyping was performed using a MegaBACE 1000 automated sequencer (GE Healthcare) following the manufacturer’s protocols. The mtDNA haplotypes generated in this study are available in GenBank under accession numbers KP724852-KP724979. STR genotypes are available in S1 Table.

### Mitochondrial DNA Data Analysis

Individual DNA sequences were assembled in Geneious v.5.4.4 [19], and the sequences of the two markers were concatenated. We also used 218 sequences from populations sampled in the Atlantic (Caribbean) and Pacific Ocean [8]. All sequences were aligned and checked by eye in Bioedit v.7.1.11 [20]. Evolutionary relationships among haplotypes were represented using a median joining network [21], as estimated in the software Network v.4.6 (www.fluxus-engeneering.com).

We used the program Arlequin 3.5 [22] to estimate standard genetic diversity indices, including Tajima’s D [23] and Fu’s F_S_ [24] neutrality tests. This program was also used to quantify the level of genetic structure using F-statistics with both pairwise colony comparisons and a hierarchical Analysis of Molecular Variance (AMOVA) based on oceanic features: Galapagos; Panama (Isla Iguana—Pacific Ocean); Caribbean (Barbuda, Dry Tortuga, Bahamas, British Virgin Islands, Belize HC, Belize MW, Jamaica, Little Cayman) and French Guiana (Grand Connétable); and Brazil (Abrolhos, Alcatrazes, Cabo Frio, Cagarras, Currais, Moleques do Sul). All genetic structure values were calculated using both traditional (F_ST_) or distance-based (Φ_ST_) F-statistics and 1,000 permutations to access the statistical significance.

### Microsatellite Data Analysis

STR genotyping was performed using the software Genetic Profiler v.2.2 (GE Healthcare). All genotypes that could not be called with certainty were repeated based on an independent PCR. We used the software Micro-checker v.2.2.3 [25] to test for null alleles and evidence for genotype miscalling at specific loci. We conducted Hardy-Weinberg equilibrium (HWE) and Linkage Disequilibrium (LD) tests in the programs Genepop [26] and Arlequin 3.5, respectively, adjusting for multiple tests using the Bonferroni correction. Whenever Micro-checker suggested genotyping problems, all genotypes were thoroughly reviewed. In addition, some individuals were genotypes multiple times to ensure the quality of our genotyping protocol. In all cases, the same genotype was called based on independent PCR amplifications. We also used Arlequin 3.5 for estimating observed and expected heterozygosity. We estimate a measure of inbreeding, F_IS_, and several genetic diversity estimates, such as the number of alleles, allelic richness, and gene diversity, in the software FSTAT [27].

To quantify the genetic structure using STR markers, we estimated pairwise F_ST_ and R_ST_ values in Arlequin 3.5 with 1,000 permutations to access statistical significance. Because we did not have STR genotypes for the Caribbean and Pacific populations studies by Hailer *et al*. [8], we used AMOVA to compare two groups: one consisting of Barbuda and Grand Connétable and the other containing all populations off the Brazilian coast. We also evaluated genetic clustering using the Bayesian method implemented in STRUCTURE v.2.3.3 [28]. The most probable number of genetic clusters, K, was determined based on the probability of data conditioned on K, as suggested in the program manual, and based on ΔK, as described in Evanno *et al*. [29]. We performed five runs for every K (from K = 1 to K = 5) using the sampled populations in the locprior model, an admixture model with correlated allele frequencies, and a burn-in of 20,000 steps, with 200,000 further steps using Markov chain Monte Carlo (MCMC). Each sampling site was considered as a different population in the analysis using the locprior option. We also performed shorter runs using different settings (no-admixture model or without locprior) to evaluate the importance of model choice, and all were consistent with the results obtained using the locprior and admixture models. The strength of recent migration among populations was estimated using BayesAss v.1.3 [30], which evaluates gene flow using a model that does not assume migration-drift equilibrium. We ran independent replicates to check for consistency using 500,000 steps as burnin followed by 50,000,000 further MCMC steps, sampling every 1,000 steps, with initial values for allele frequencies, migration rates, and inbreeding adjusted for adequate mixing among chains. Migration was considered statistically significant whenever the 95% lower bound for the estimate (considering 1.96xSD [standard deviation]) was larger than zero. Population size estimates for each population individually were obtained using the STR markers in the program Lamarc [31] assuming a mutation rate of and 0.0002/locus/year (0.0001–0.0004) for STRs [32]–[34] and a generation time of 15 years based on estimates of survival and sexual maturity for frigatebirds [35]. We used the likelihood criterion and a search strategy based two replicates consisting of 20 short chains of 5,000 steps and 2 long chains of 500,000 steps.

## Results

### Mitochondrial DNA Data

**Fig 2:**
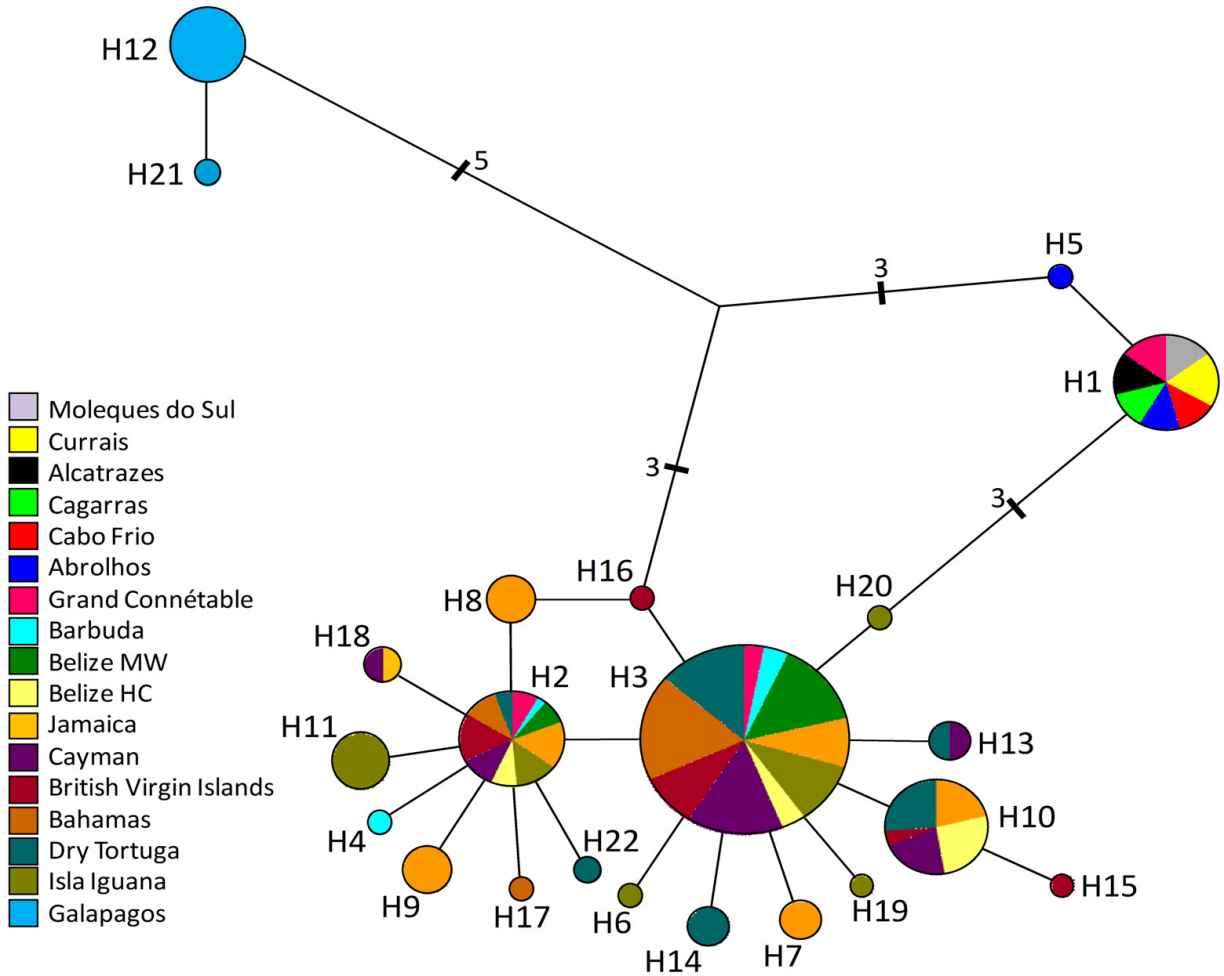
Median-joining network of mtDNA haplotypes. The size of the circles is proportional to haplotype frequency in the sample. Haplotypes from Brazil are represented in light grey, from Grand Connétable in a hatching pattern, and from Barbuda in black. All haplotypes found in Caribbean populations (except Barbuda) are represented in white color (including Isla Iguana; for details see Hailer et al., 2010). Connectors represent one mutation step except when noted.

Considering both mtDNA genes sequenced in this study, we analyzed 1227 bp and found 26 variable sites. The total number of haplotypes was 22, and the haplotype diversity for the entire sample was 0.726 (Standard Deviation 0.022). Detailed information about the haplotype composition for each population is shown in S2 Table. The Median-Joining haplotype network is shown in Fig 2. Of note, the Caribbean population showed higher diversity when compared to the Brazilian sample. All Brazilian individuals (n = 41) had the same haplotype, except for a single individual from the Abrolhos population. Some individuals from Grand Connétable, in northern South America, had the same haplotype observed in Brazilian samples, indicating gene flow or shared ancestral polymorphism. The other haplotypes found in the Grand Connétable population were shared with the populations from the Caribbean, including Barbuda.

A hierarchical AMOVA considering Φ-statistics and four groups (Galapagos; Pacific Ocean; Caribbean and French Guiana; Brazil) resulted in overall high levels of genetic structure among the groups, whereas traditional F-statistics resulted in smaller values (Table 1). Because Φ-statistics take into account mutational distance and the Galapagos population has highly divergent haplotypes, we performed AMOVA with three groups, excluding the Galapagos population. The results were very similar (Table 1), possibly reflecting the low haplotype sharing between Brazil and the other groups. Table 2 shows pairwise values for Φ_ST_ and F_ST_ among the populations. As expected, the Brazilian populations are not significantly different among each other. Overall, the different Caribbean populations also do not show a statistically significant genetic structure among them or compared to Isla Iguana. In contrast, the Grand Connétable population exhibited significant differentiation from some of the populations from Brazil and the Caribbean, in agreement with its haplotype composition.

**Table 1:** AMOVA Based on mtDNA data.

	Four groups^1^		Three groups^2^	
Level	Φ-statistics	F-statistics	Φ-statistics	F-statistics
Among groups	0.831	0.488	0.737	0.440
Among populations within groups	0.027	0.042	0.039	0.042
Within populations	0.142	0.470	0.224	0.518

^1^[Galapagos; Pacific; Caribbean; Brazil].

^2^[Pacific; Caribbean; Brazil].

See text for details.

**Table 2:** Pairwise F_ST_ (upper diagonal) and Φ_ST_ (lower diagonal) values for mtDNA data.

Population	1	2	3	4	5	6	7	8	9	10	11	12	13	14	15	16	17
1- Moleques do Sul		0.000	0.000	0.000	0.000	0.020	**0.251**	**0.755**	**0.595**	**0.621**	**0.773**	**0.587**	**0.586**	**0.824**	**0.461**	**0.560**	**0.926**
2- Currais	0.000		0.000	0.000	0.000	0.038	**0.267**	**0.769**	**0.605**	**0.629**	**0.779**	**0.596**	**0.600**	**0.829**	**0.470**	**0.570**	**0.928**
3- Alcatrazes	0.000	0.000		0.000	0.000	0.000	0.233	**0.738**	**0.584**	**0.613**	**0.767**	**0.578**	**0.570**	**0.818**	**0.451**	**0.550**	**0.923**
4- Cagarras	0.000	0.000	0.000		0.000	-0.024	0.213	**0.719**	**0.571**	**0.604**	**0.761**	**0.568**	**0.552**	**0.812**	**0.439**	**0.538**	**0.920**
5- Cabo Frio	0.000	0.000	0.000	0.000		-0.024	0.213	**0.719**	**0.571**	**0.604**	**0.761**	**0.568**	**0.552**	**0.812**	**0.439**	**0.538**	**0.920**
6- Abrolhos	0.020	0.038	0.000	0.020	0.020		0.126	**0.595**	**0.498**	**0.539**	**0.700**	**0.502**	**0.464**	**0.742**	**0.370**	**0.468**	**0.844**
7- Grand Connétable	**0.351**	**0.367**	**0.333**	0.313	0.313	**0.325**		**0.294**	**0.387**	**0.428**	**0.475**	**0.418**	**0.369**	**0.458**	**0.373**	**0.171**	**0.643**
8- Barbuda	**0.920**	**0.926**	**0.914**	**0.907**	**0.907**	**0.885**	**0.207**		-0.039	-0.050	-0.040	-0.028	0.100	-0.025	0.034	-0.040	**0.771**
9- British Virgin Is.	**0.872**	**0.876**	**0.868**	**0.863**	**0.863**	**0.856**	**0.174**	-0.055		0.000	0.009	0.022	0.055	0.081	0.019	-0.018	**0.631**
10- Cayman	**0.872**	**0.876**	**0.869**	**0.865**	**0.865**	**0.860**	**0.238**	-0.012	-0.010		0.007	-0.021	0.071	0.031	0.053	0.010	**0.646**
11- Bahamas	**0.932**	**0.934**	**0.930**	**0.928**	**0.928**	**0.920**	**0.375**	-0.010	0.064	0.021		**0.057**	**0.265**	-0.037	**0.165**	0.062	**0.777**
12- Dry Tortuga	**0.858**	**0.861**	**0.854**	**0.850**	**0.85**	**0.845**	**0.223**	0.023	0.022	-0.018	0.038		0.049	**0.068**	**0.046**	0.024	**0.616**
13- Belize HC	**0.880**	**0.885**	**0.874**	**0.867**	**0.867**	**0.857**	**0.182**	0.084	0.045	0.035	**0.190**	0.026		**0.289**	0.003	0.082	**0.636**
14- Belize MW	**0.956**	**0.957**	**0.954**	**0.953**	**0.953**	**0.940**	**0.399**	0.024	0.022	0.003	-0.033	0.027	**0.208**		**0.101**	0.076	**0.819**
15- Jamaica	**0.797**	**0.801**	**0.791**	**0.786**	**0.786**	**0.785**	**0.125**	-0.018	-0.001	**0.059**	**0.090**	**0.091**	0.051	**0.179**		0.030	**0.498**
16- Isla Iguana	**0.849**	**0.853**	**0.845**	**0.840**	**0.840**	**0.835**	**0.381**	-0.047	-0.004	0.040	0.030	**0.077**	**0.118**	0.043	0.029		**0.596**
17- Galapagos	**0.992**	**0.992**	**0.991**	**0.991**	**0.991**	**0.983**	**0.877**	**0.970**	**0.950**	**0.949**	**0.972**	**0.943**	**0.956**	**0.980**	**0.914**	**0.941**	

Values in bold are statistically significant (P<0.05).

### Microsatellite Loci Data

All loci were in linkage equilibrium and in HWE, except for Fmin17, which showed significant excess homozygosity. However, all other loci had some degree of homozygote excess (which could be an indication of inbreeding). Genotyping of some individuals based on independent PCR amplifications of Fmin17 showed consistent results, and we have thus decided to retain this locus in the main dataset and test how this could affect our results and conclusions. S1 Table shows all genotypes for the individuals used in this study. Table 3 summarizes the genetic diversity for STR loci over the studied populations considering all loci (see S3 Table for the results excluding locus Fmin17). We found between 5 and 23 alleles per locus. The allelic richness, number of alleles, and observed and expected heterozygosity were highest in Barbuda, followed by Grand Connétable and then by the Brazilian populations. Considering all loci, the F_IS_ values were distributed between 0.039 (Abrolhos) and 0.231 (Moleques do Sul) (Table 2). As expected, excluding Fmin17 resulted in lower values, even though the Abrolhos and Moleques do Sul populations still showed the lowest and highest values (-0.028 and 0.155), respectively (S3 Table).

**Table 3:** Genetic diversity values for STR data.

Population	N	N_A_	AR	F_IS_	H_O_	H_E_
Barbuda	29	8.13	5.219	0.102	0.630 ± 0.243	0.700 ± 0.231
Grand Connétable	37	7.13	4.681	0.131	0.583 ± 0.191	0.669 ± 0.247
Abrolhos	18	5.13	3.910	0.039	0.556 ± 0.288	0.578 ± 0.228
Cabo Frio	14	4.25	3.470	0.106	0.471 ± 0.238	0.524 ± 0.228
Cagarras	9	4.00	3.819	0.206	0.535 ± 0.242	0.581 ± 0.265
Alcatrazes	18	5.00	3.825	0.151	0.503 ± 0.233	0.591 ± 0.247
Currais	9	4.13	3.812	0.159	0.543 ± 0.270	0.560 ± 0.288
Moleques do Sul	22	4.88	3.595	0.231	0.415 ± 0.264	0.537 ± 0.248

N—sample size; N_A_—Average number of alleles; AR—Allelic richness; H_O_—Observed heterozygosity ± standard deviation; H_E_—Expected heterozygosity ± standard deviation.

The measures of genetic structure between the Brazilian populations vs. Barbuda (Caribbean) + Grand Connétable (French Guiana) were small and similar using either R-statistics or F-statistics, resulting in an “among group” component of 3.21% and 6.43%, respectively. The “among populations within group” component was 3.54% and 2.51%, and the “within population” component estimates were 93.31% and 91.05%. The pairwise R_ST_ and F_ST_ values for the STR dataset are shown in Table 4. In general, there was evidence of significant structure between Barbuda and most of the Brazilian populations and between Grand Connétable and a few Brazilian populations. Interestingly, there were also a few significant though small values among the Brazilian populations. Again, excluding the Fmin17 locus did not result in a significant change in these results. In the AMOVA analysis, the “among group” values were 2.77% and 5.78% for R-statistics and F-statistics, respectively; in the pairwise matrix, ten out of 14 values remained significant (S4 Table).

**Table 4:** Pairwise F_ST_ (upper diagonal) and R_ST_ (lower diagonal) values for STR data.

Population	1	2	3	4	5	6	7	8
1- Abrolhos		<0.001	0.001	<0.001	0.004	0.003	<0.001	<0.001
2- Cabo Frio	**0.088**		-0.004	-0.003	-0.001	-0.004	**0.004**	<0.001
3- Cagarras	0.001	<0.001		**0.005**	0.010	0.006	**0.006**	**0.005**
4- Alcatrazes	0.017	0.068	-0.020		0.004	0.003	0.001	-0.001
5- Currais	-0.039	<0.001	-0.020	0.022		0.004	**0.003**	0.003
6- Moleques do Sul	**0.085**	-0.021	0.019	**0.072**	0.066		**0.003**	0.002
7- Barbuda	**0.036**	**0.138**	0.040	**0.063**	0.005	**0.159**		<0.001
8- Grand Connétable	-0.021	**0.060**	-0.014	0.011	-0.059	**0.084**	0.017	

Values in bold are statistically significant (P<0.05).

**Fig 3:**
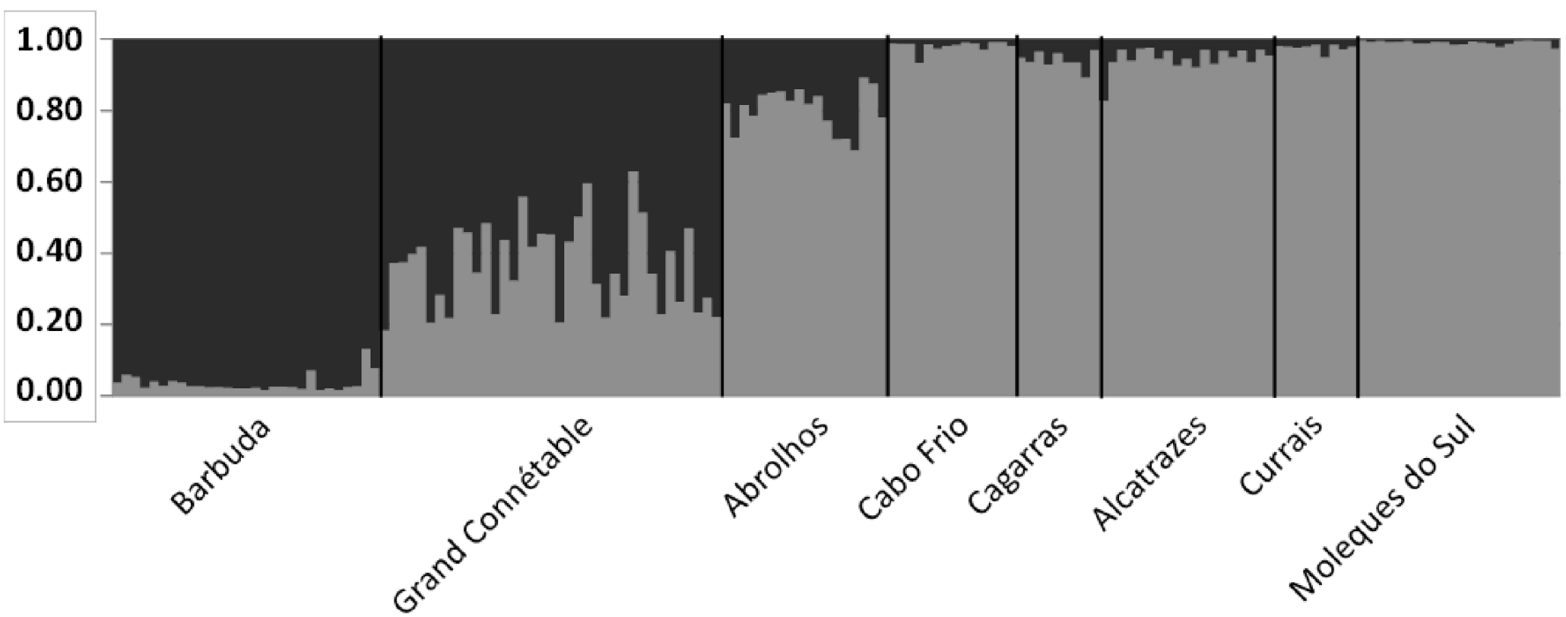
Ancestry estimates for all individuals based on K = 2 genetic clusters.

Despite these low genetic structure values, the Bayesian clustering analysis performed in STRUCTURE found K = 2 as the best number of genetic populations supported by the STR data. The posterior probability of data conditioned at K = 2 was ~1.0; for K = 3, the second best K-value, the posterior probability was 5.6x10^-33^, while K = 1 had a posterior probability of only 1.5x10^-85^. The ΔK approach also supported two genetic clusters (S1 Fig). Fig 3 shows the individual probabilities of assignment for each K value: one genetic cluster is clearly associated with Barbuda, and the other is associated with the Brazilian populations. Grand Connétable had a higher component of the first cluster but also showed a significant portion of the second cluster. Abrolhos, the northernmost Brazilian population analyzed in this study, had a major component in the second cluster while still maintaining some association in the first cluster. Again, these results are robust with the exclusion of Fmin17 from the analysis (S2 Fig), as well as to the usage of the locprior option and the no-admixture ancestry model (S5 Table). Estimates of recent migration based on Bayesian population assignment showed that there are migratory connections from Moleques do Sul to all other Brazilian populations, except Abrolhos, from Abrolhos to all other Brazilian populations, except Moleques do Sul, and from Abrolhos to Grand Connétable (S6 Table). Interestingly, we did not detect migration between Barbuda and Grand Connétable, and all inferred migrations were unidirectional. The estimates of the effective population size (N_E_) in Lamarc showed a clear distinction between Barbuda and Grand Connétable, with larger numbers on the one hand, and the Brazilian populations, with lower and similar values on the other hand (Table 5).

**Table 5:** Demographic estimates based on coalescent analysis.

Population	N_E_ (95% CI)
Barbuda	1,021 (905–1,159)
Grand Connétable	906 (823–998)
Abrolhos	426 (371–491)
Cabo Frio	296 (243–365)
Cagarras	370 (299–464)
Alcatrazes	330 (283–390)
Currais	334 (267–425)
Moleques do Sul	360 (318–410)

95%CI– 95% Credible interval. All estimates assuming 15 years for generation time (see text for details).

## Discussion

### Genetic structure in the Magnificent Frigatebird

There was no mtDNA haplotype sharing between the Brazilian and Caribbean populations, indicating, at least based on mtDNA, that these populations are effectively isolated (Fig 2, Tables 1 and 2). Grand Connétable, off French Guiana, seems to be a key population, as this population shares mtDNA haplotypes with both the Caribbean and Brazilian populations. The results based on STR markers support the inferences based on mtDNA: a general low level of genetic structure among the populations (Tables 3 and 4, S3 and S4 Tables). Nonetheless, the strongest genetic structure detected in our data separates Barbuda from the Brazilian populations, reinforcing the “intermediate” nature of the Grand Connétable population (Fig 3 and S2 and S5 Tables). Taken together, our results suggest that the western Atlantic populations off Brazil represent a third evolutionary lineage in this species, together with 1) populations from the Caribbean and coastal Pacific, and 2) Galápagos, which is a population clearly isolated from these [8].

We were able to identify two major patterns of recent migratory connections (S6 Table), even though the small values for genetic structure in our dataset reduced the statistical power for detecting migration at a fine scale. Moleques do Sul, the southernmost Brazilian population, sends migrants to all the other Brazilian populations except Abrolhos. On the other hand, Abrolhos sends migrants to Grand Connétable and to all the other Brazilian populations except Moleques do Sul. This raises the question if this species may disperse over wide land areas. Indeed, González-Jaramillo & Rocha-Olivares [36] found that Mexican populations of *F*. *magnificens* from the Pacific or Atlantic coast share mtDNA haplotypes, and Hailer *et al*. [8] found no genetic structure between populations from the Caribbean and the Pacific Coast of Mexico, Central, and Northern South America using a wide set of genetic markers. Taken together, these studies suggest that Mexico and Central America may not constrain dispersal between populations from the Caribbean Sea and coastal Pacific Oceans. Therefore, *F*. *magnificens* populations may maintain gene flow even when separated by wide landmasses. However, another study found a low differentiation (Φ_ST_ = 0.048) between *F*. *magnificens* colonies from Mexican Pacific and the Caribbean, and did not observe birds flying between these areas based on mark-resight data [37].

The lack of genetic structure among Caribbean/Pacific and among Brazilian populations is unsurprising for a seabird species with a wide foraging range such as the Magnificent Frigatebird, which can easily cover distances between colonies in less than one day. Studies in both *F*. *magnificens* [8], [36], [37], and *F*. *minor* [38], [39] are unanimous in suggesting that isolation by distance is absent in these birds, and that other factors, including behavioral and ecological traits may be responsible for genetic structure among colonies. In a review of several seabird taxa, Friesen *et al*. [40] found a vast array of results, varying from virtual panmixia (*e*.*g*., Grey-headed Albatross *Thalassarche chrysostoma*) to genetic structure in the absence of obvious barriers to gene flow (*e*.*g*., Galapagos Petrel *Pterodroma phaeopygia* and Xantu’s Murrelet *Synthliboramphus hypoleucus*). However, most species exhibited detectable genetic structure across their ranges, especially those with distributions fragmented by land or ice [40]. These results are also in agreement to those discussed previously in that geographic distance itself may promote population differentiation in seabirds when coupled to ecological, behavioral and historical differences such as breeding distribution and population-specific nonbreeding seasons [40]. Abbot and Double [41], for example, reported different patterns of genetic structure in taxonomically closely related species of albatrosses. These authors found no genetic structure among populations of White-capped Albatross *Thalassarche steadi* but significant structure among populations of Shy Albatross *T*. *cauta*. A study with a seabird breeding on Brazilian Islands, the South American Tern *Sterna hirundinacea*, found low genetic structure for both mtDNA (F_ST_ between 0.0005 and 0.0184) and microsatellites (F_ST_ between -0.0038 and 0.0938) [42], in agreement to the low genetic structure among Brazilian populations found for *F*. *magnificens*.

Among several possible ecological or environmental differences that could explain the patterns found in our study, our results are compatible with a strong role of major oceanic winds in shaping the principal patterns of genetic structure. For example, the strong genetic structure between Brazil *vs*. Caribbean + Grand Connétable may be associated with the influence of the South Atlantic anticyclone over the coast of South America. This atmospheric high-pressure system is centered at 25°S, 15°W and is the source of the warm air masses and northeasterly winds that prevail along most of the eastern coast of Brazil; these winds are stronger during summer in the Southern Hemisphere, January—March. The northern or equatorial coast of Brazil is roughly found within the Intertropical Convergence Zone, the region near the Equator of converging trade winds of both the Northern and Southern Hemispheres and upward air currents [43], [44].

In equatorial latitudes, Magnificent Frigatebirds take advantage of the uprising air columns, or thermals, that are produced by heating of the ocean surface due to strong solar radiation [43]. Therefore, frigatebirds circle upwards on a thermal and then soar from one thermal to another over long distances [45]. Based on the atmospheric circulation, it is thus expected that the dispersal of Magnificent Frigatebirds from the eastern Brazilian colonies to equatorial latitudes are constrained, though apparently not fully inhibited, by the prevailing northeasterly winds. The reverse movement; *i*.*e*., from the North Atlantic (Caribbean + Grand Connétable) to Brazil, is also disfavored but not impeded by the easterly trade winds and calms of the Intertropical Convergence Zone. Indeed, Weimerskirch et al. [45] reported two tracked individuals from Grand Connétable that moved westward along South American coastline, and hence against surface winds; one of them reached as far west as Trinidad, located 1,400 km from its breeding colony. Even though some caution must be taken when interpreting results from telemetry because the use of a wide range does not automatically imply in gene flow (*e*.*g*. [38]), the “Caribbean” ancestry found in Abrolhos, for example, may reflect some gene flow among different colonies against the most favored wind routes.

Connections among Brazilian populations may also be explained by the influence of atmospheric circulation. During the Southern Hemisphere winter, northeasterly winds that blow along the eastern Brazilian coast become weaker thus often are replaced by the gale- and storm-force southerly winds from the Polar anticyclone. Cold air masses from these anticyclones move into south Brazil, causing rainy, cold, and windy days. Such a phenomenon is stronger in south Brazil but may reach 23μS in the State of Rio de Janeiro and less frequently 10μS in the State of Bahia [44]. When southerly winds are blowing, southern ocean seabirds normally fly north along Brazil south and southeast coasts, and sometimes tropical latitudes [2]. Thus, the relative importance of oceanic wind patterns may be associated with our findings of Moleques do Sul being the source of migrants for all populations, except Abrolhos, and vice-versa. This may also explain why Moleques do Sul shows the highest F_IS_ estimates (Table 3, S3 Table).

### Demographic Scenarios

We found a clear distinction between the level of genetic variation in the Caribbean and in Brazil for both mtDNA and STR (Fig 2, Table 3, S4 Table). For instance, the only Caribbean population sampled by us (Barbuda) had more mtDNA haplotypes than all the Brazilian populations considered together (Fig 2). This difference reflects in the estimates of historical effective population sizes for these populations (Table 5). Several analyses for both mtDNA and STR suggested that Grand Connétable has a genetic profile that can be seen as “intermediate” between the extremes of Barbuda and Southern Brazilian colonies. Palacio [46] considered the Caribbean as a potential center of biodiversity based on the early occurrence, for several taxonomic groups, of species in this region compared to later appearances in adjacent geographic areas, including the Brazilian coast. In this case, and judging from the degree of mtDNA divergence among populations, an ancient dispersal, estimated by Hailer *et al*., [8] at ~250,000 years ago, would have led to the emergence of the Galapagos population, whereas a more recent dispersal event would be associated with the colonization of Brazilian coastal islands. The larger effective population size in the Caribbean is also compatible with this region playing the role of a historical “refuge” for population diversification across the Pleistocene in repeated “out of the Caribbean” dispersal episodes.

## Supporting Information

S1 Fig ΔK estimate of the best number of genetic clusters in Structure. (PDF)

S2 Fig Bayesian ancestry estimates based on K = 2 excluding locus Fmin17. (PDF)

S1 Table Genotypes observed for all sampled individuals. (PDF)

S2 Table Haplotype diversity and distribution in all sampled populations. (PDF)

S3 Table Genetic diversity values for STR data excluding locus Fmin17. (PDF)

S4 Table Pairwise F_ST_ and R_ST_ for STR data excluding locus Fmin17. (PDF)

S5 Table Structure analysis for K = 2 based on different assumptions. (PDF)

S6 Table Migration rate estimates using BayesAss. (PDF)
