# Supplementary material for: Population Genetic Structure of the Magnificent Frigatebird Fregata magnificens (Aves, Suliformes) Breeding Colonies in the Western Atlantic Ocean
Source: PLoS One. 2016 Feb 22;11(2):e0149834. doi: 10.1371/journal.pone.0149834 (PMC4762693; doi:10.1371/journal.pone.0149834)
Supplement: S2 Fig — (PDF) [file pone.0149834.s002.pdf]

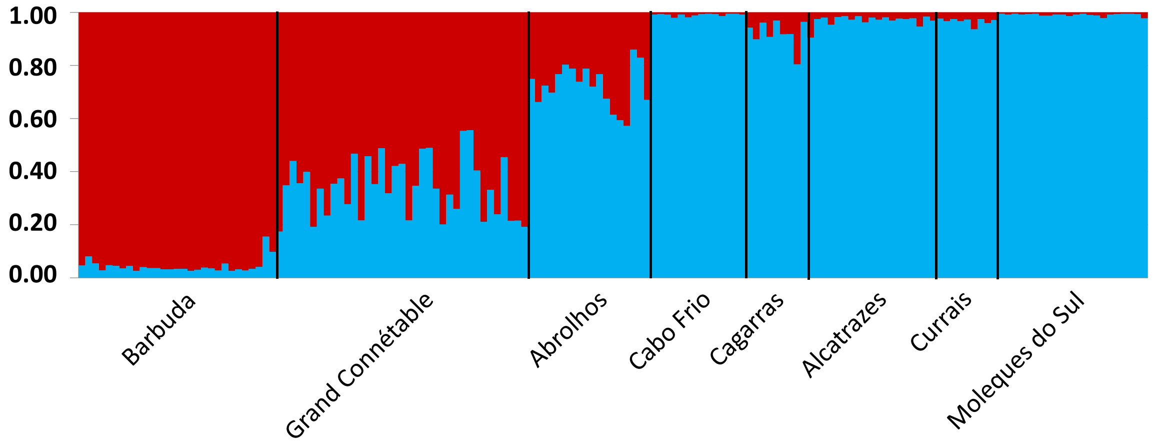

S2 Figure. Bayesian ancestry estimates for all individuals based on K=2 genetic clusters, excluding locus Fmin17.
