# Supplementary material for: Population Genetic Structure of the Magnificent Frigatebird Fregata magnificens (Aves, Suliformes) Breeding Colonies in the Western Atlantic Ocean
Source: PLoS One. 2016 Feb 22;11(2):e0149834. doi: 10.1371/journal.pone.0149834 (PMC4762693; doi:10.1371/journal.pone.0149834)
Supplement: S1 Table — (PDF) [file pone.0149834.s003.pdf]

**S1 Table.** Genotypes observed for all sampled individuals

| Population | Sample | STR Loci | | | | | | | | | | | | | | | |
|---|---|---|---|---|---|---|---|---|---|---|---|---|---|---|---|---|---|
| | | Fmin02 | | Fmin11 | | Fmin12 | | Fmin14 | | Fmin15 | | Fmin16 | | Fmin17 | | Fmin18 | |
| Barbuda | FMCA05 | 202 | 202 | 178 | 180 | 157 | 165 | 175 | 179 | 226 | 234 | 160 | 160 | ? | ? | 214 | 230 |
| | FMCA06 | 204 | 206 | 178 | 180 | 163 | 175 | 175 | 183 | 214 | 230 | 160 | 160 | 432 | 432 | 206 | 210 |
| | FMCA07 | 204 | 224 | 180 | 180 | 173 | 177 | 179 | 191 | 230 | 230 | 160 | 160 | 420 | 428 | 214 | 222 |
| | FMCA08 | 208 | 212 | ? | ? | 151 | 155 | 175 | 179 | 230 | 238 | 160 | 160 | ? | ? | ? | ? |
| | FMCA102 | 204 | 232 | 178 | 180 | 157 | 173 | 179 | 191 | 218 | 226 | 156 | 160 | ? | ? | 218 | 218 |
| | FMCA103 | 204 | 204 | 178 | 184 | 177 | 177 | 179 | 183 | 218 | 222 | 160 | 160 | 432 | 432 | 222 | 222 |
| | FMCA104 | 206 | 224 | 178 | 178 | 163 | 175 | 179 | 187 | 222 | 230 | 160 | 160 | 424 | 432 | 206 | 214 |
| | FMCA127 | 200 | 212 | 178 | 180 | 157 | 163 | 179 | 187 | 218 | 226 | 160 | 160 | 420 | 432 | ? | ? |
| | FMCA128 | 208 | 230 | 178 | 178 | 157 | 163 | 171 | 179 | 218 | 222 | 160 | 176 | 424 | 432 | 206 | 214 |
| | FMCA129 | 204 | 224 | 180 | 180 | 157 | 163 | 179 | 179 | 222 | 230 | 160 | 180 | 432 | 432 | 206 | 206 |
| | FMCA130 | 238 | 240 | 178 | 180 | 157 | 179 | 179 | 179 | 218 | 226 | 160 | 172 | 432 | 432 | 214 | 242 |
| | FMCA131 | 226 | 242 | 180 | 180 | 157 | 163 | 175 | 195 | 206 | 230 | 160 | 160 | 432 | 432 | 202 | 214 |
| | FMCA132 | 206 | 206 | 178 | 180 | 163 | 165 | 175 | 187 | 226 | 230 | 160 | 160 | 432 | 432 | ? | ? |
| | FMCA133 | ? | ? | 178 | 180 | 157 | 163 | 183 | 191 | 222 | 230 | 160 | 172 | 432 | 432 | 210 | 210 |
| | FMCA134 | 214 | 242 | 178 | 178 | 163 | 173 | 175 | 183 | 222 | 222 | 160 | 160 | 432 | 432 | 210 | 242 |
| | FMCA135 | 212 | 216 | 178 | 178 | 165 | 173 | ? | ? | ? | ? | 160 | 160 | 432 | 432 | 206 | 214 |
| | FMCA136 | 208 | 208 | 178 | 180 | 165 | 179 | 175 | 179 | 222 | 226 | 156 | 160 | 432 | 436 | 218 | 218 |
| | FMCA137 | 216 | 216 | 178 | 180 | 157 | 173 | 183 | 187 | 218 | 218 | 160 | 160 | 420 | 432 | 210 | 210 |
| | FMCA138 | 206 | 208 | 178 | 180 | 157 | 173 | 175 | 183 | 222 | 230 | 160 | 160 | 436 | 436 | 202 | 206 |
| | FMCA139 | 208 | 224 | 178 | 180 | 157 | 163 | 187 | 191 | 226 | 230 | 160 | 160 | 428 | 428 | 214 | 234 |
| | FMCA140 | 208 | 210 | 178 | 180 | 165 | 177 | 179 | 187 | 230 | 238 | 160 | 160 | 428 | 428 | ? | ? |
| | FMCA141 | 208 | 228 | 178 | 180 | 175 | 175 | 179 | 187 | 218 | 226 | 160 | 160 | 424 | 432 | 214 | 214 |
| | FMCA142 | 210 | 220 | 178 | 178 | 157 | 173 | 183 | 183 | 222 | 226 | 160 | 172 | 436 | 436 | 206 | 206 |
| | FMCA143 | 210 | 214 | 180 | 180 | 157 | 173 | 175 | 179 | 218 | 218 | 160 | 160 | 432 | 436 | 206 | 206 |
| | FMCA144 | 212 | 212 | 178 | 178 | 157 | 157 | 175 | 175 | 234 | 234 | 160 | 160 | 432 | 432 | 210 | 222 |
| | FMCA38 | 206 | 206 | 178 | 180 | 157 | 165 | 175 | 179 | 218 | 230 | 160 | 160 | 428 | 428 | 218 | 222 |
| | FMCA39 | 204 | 230 | 178 | 180 | 157 | 163 | 175 | 191 | 222 | 238 | 160 | 160 | 432 | 432 | 214 | 214 |
| | FMCA50 | 202 | 204 | 178 | 180 | 149 | 173 | 179 | 179 | 218 | 222 | 160 | 160 | 428 | 428 | 206 | 230 |
| | FMCA51 | 204 | 224 | 178 | 178 | 149 | 173 | 171 | 179 | 206 | 222 | 160 | 160 | 428 | 428 | 210 | 218 |
| Grand Connétable | 14A | 202 | 214 | 178 | 180 | 159 | 159 | 175 | 179 | 218 | 218 | 160 | 160 | 420 | 432 | 206 | 206 |
| | 15A | 206 | 234 | 180 | 180 | 157 | 173 | 179 | 191 | 214 | 222 | 160 | 160 | 420 | 428 | ? | ? |
| | 16A | 202 | 204 | 178 | 180 | 149 | 159 | ? | ? | ? | ? | 160 | 160 | 432 | 432 | 214 | 214 |

| | | | | | | | | | | | | | | | | | |
|---|---|---|---|---|---|---|---|---|---|---|---|---|---|---|---|---|---|
| | 17A | ? | ? | 178 | 178 | 173 | 177 | 179 | 183 | 214 | 230 | 160 | 160 | 424 | 432 | 206 | 214 |
| | 18A | 200 | 210 | 180 | 180 | 173 | 173 | 171 | 183 | 222 | 230 | 160 | 160 | 420 | 428 | 210 | 230 |
| | 1A | 204 | 212 | 178 | 180 | 157 | 167 | 171 | 183 | 218 | 218 | 156 | 160 | 420 | 428 | ? | ? |
| | 20A | 210 | 228 | 178 | 180 | 173 | 173 | 175 | 175 | 230 | 234 | 160 | 160 | 432 | 432 | ? | ? |
| | 21A | ? | ? | ? | ? | 157 | 167 | 171 | 179 | 230 | 230 | 160 | 160 | 428 | 428 | 206 | 210 |
| | 22A | ? | ? | 180 | 180 | 149 | 163 | 179 | 183 | 214 | 230 | 160 | 160 | 424 | 424 | 206 | 206 |
| | 23A | ? | ? | ? | ? | 149 | 163 | 179 | 179 | 218 | 218 | 160 | 160 | 420 | 420 | ? | ? |
| | 25A | ? | ? | ? | ? | 157 | 175 | 171 | 187 | 206 | 214 | 160 | 160 | 420 | 420 | 210 | 210 |
| | F10 | 204 | 204 | 178 | 180 | ? | ? | 175 | 179 | 230 | 230 | 160 | 160 | 420 | 428 | ? | ? |
| | F11 | ? | ? | 178 | 180 | 165 | 173 | 183 | 187 | 218 | 218 | 160 | 160 | 420 | 432 | 206 | 214 |
| | F2 | 200 | 204 | 178 | 180 | ? | ? | 171 | 179 | 230 | 230 | 160 | 160 | 416 | 420 | ? | ? |
| | F3 | 200 | 226 | 178 | 180 | 157 | 173 | 171 | 187 | 214 | 238 | 160 | 160 | 428 | 428 | 214 | 218 |
| | F4 | 204 | 204 | 180 | 180 | 149 | 149 | 179 | 183 | 206 | 222 | 160 | 160 | 420 | 420 | 206 | 210 |
| | F5 | 206 | 220 | 178 | 180 | ? | ? | 179 | 183 | 222 | 234 | ? | ? | 420 | 420 | ? | ? |
| | F6 | 204 | 216 | 180 | 180 | 149 | 173 | 179 | 179 | 218 | 218 | 160 | 160 | 424 | 432 | 206 | 214 |
| | F7 | 200 | 240 | 178 | 180 | 149 | 173 | 171 | 175 | 214 | 218 | 160 | 160 | 424 | 432 | 206 | 214 |
| | F9 | 206 | 206 | 178 | 180 | 157 | 173 | 171 | 175 | 218 | 230 | 160 | 160 | 428 | 428 | 206 | 206 |
| | FMGC40 | 206 | 212 | 180 | 180 | 173 | 173 | 179 | 183 | 214 | 222 | 160 | 160 | 420 | 420 | 206 | 206 |
| | FMGC41 | 204 | 204 | 178 | 180 | 149 | 173 | 171 | 183 | 214 | 222 | 160 | 172 | 424 | 432 | 218 | 226 |
| | FMGC44 | 206 | 230 | 178 | 180 | 149 | 157 | 179 | 179 | 214 | 230 | 160 | 160 | 424 | 424 | 214 | 214 |
| | FMGC52 | 230 | 246 | 180 | 180 | 149 | 173 | 179 | 191 | 218 | 230 | 160 | 160 | 428 | 428 | 210 | 214 |
| | FMGC53 | 202 | 228 | ? | ? | ? | ? | 191 | 191 | ? | ? | 160 | 160 | 420 | 428 | 206 | 206 |
| | FMGC54 | 202 | 228 | 178 | 180 | 173 | 173 | 179 | 183 | 218 | 238 | 160 | 160 | 420 | 428 | 206 | 214 |
| | FMGC55 | 204 | 216 | 180 | 180 | 157 | 175 | 179 | 183 | 218 | 238 | 160 | 172 | 420 | 428 | 210 | 210 |
| | FMGC56 | 206 | 206 | 178 | 180 | 173 | 173 | 179 | 187 | 214 | 214 | 160 | 160 | 420 | 420 | 210 | 214 |
| | FMGC57 | 204 | 204 | 178 | 180 | 173 | 173 | 171 | 183 | 214 | 218 | 160 | 160 | 428 | 428 | 214 | 234 |
| | FMGC58 | 204 | 204 | 178 | 180 | 157 | 173 | 179 | 179 | 222 | 230 | 160 | 172 | 416 | 428 | 218 | 218 |
| | FMGC59 | 212 | 238 | 178 | 178 | 157 | 173 | 179 | 179 | 222 | 238 | 160 | 160 | 420 | 428 | 206 | 242 |
| | FMGC60 | 212 | 212 | 180 | 180 | 149 | 165 | 179 | 179 | 222 | 222 | 160 | 160 | 420 | 420 | ? | ? |
| | FMGC61 | 206 | 206 | 180 | 180 | 157 | 157 | 179 | 187 | 206 | 230 | 160 | 160 | 420 | 428 | ? | ? |
| | FMGC62 | 200 | 200 | 178 | 178 | 149 | 157 | 179 | 179 | 218 | 238 | 160 | 172 | 420 | 428 | 210 | 214 |
| | FMGC63 | 206 | 206 | 180 | 180 | 163 | 165 | 183 | 191 | 206 | 222 | 160 | 160 | 420 | 428 | 206 | 234 |
| | FMGC64 | 220 | 230 | 178 | 180 | 157 | 173 | 175 | 183 | 218 | 230 | 160 | 160 | 420 | 420 | 214 | 218 |
| | FMGC66 | 222 | 238 | 178 | 180 | 167 | 177 | 175 | 175 | 218 | 218 | 160 | 160 | 424 | 432 | ? | ? |
| Abrolhos | FMAB21 | 226 | 228 | 178 | 182 | 149 | 173 | 179 | 187 | 222 | 230 | 160 | 160 | 420 | 420 | 214 | 234 |
| | FMAB22 | 204 | 206 | 178 | 178 | 173 | 181 | 175 | 179 | 222 | 230 | 160 | 160 | 420 | 428 | 206 | 210 |

| | | | | | | | | | | | | | | | | | |
|---|---|---|---|---|---|---|---|---|---|---|---|---|---|---|---|---|---|
| | FMAB23 | 206 | 206 | 178 | 178 | 173 | 173 | 179 | 179 | 214 | 230 | 160 | 160 | 420 | 420 | 206 | 218 |
| | FMAB24 | 224 | 228 | 178 | 180 | 149 | 149 | 179 | 179 | 226 | 230 | 160 | 160 | 420 | 420 | 210 | 222 |
| | FMAB25 | ? | ? | 178 | 178 | 173 | 173 | ? | ? | 214 | 230 | 160 | 160 | 420 | 420 | 214 | 218 |
| | FMAB26 | ? | ? | 178 | 180 | 173 | 173 | 179 | 179 | 230 | 234 | 160 | 160 | ? | ? | 210 | 214 |
| | FMAB27 | 202 | 204 | 178 | 178 | 149 | 173 | 179 | 179 | 218 | 230 | 160 | 160 | 420 | 420 | 210 | 214 |
| | FMAB29 | 204 | 206 | 178 | 180 | 149 | 173 | 179 | 183 | 222 | 226 | 160 | 164 | 424 | 424 | 210 | 210 |
| | FMAB30 | 210 | 228 | 178 | 180 | 173 | 173 | 179 | 183 | 214 | 214 | 160 | 160 | 424 | 424 | 210 | 214 |
| | FMAB31 | 204 | 226 | 180 | 180 | 149 | 157 | 179 | 179 | 222 | 230 | 160 | 160 | 424 | 424 | 206 | 214 |
| | FMAB74 | 204 | 204 | 180 | 180 | 173 | 173 | 179 | 183 | 226 | 230 | 160 | 160 | 420 | 420 | 214 | 222 |
| | FMAB75 | 206 | 206 | 180 | 180 | 149 | 173 | 179 | 187 | 214 | 226 | 160 | 172 | 420 | 420 | 206 | 214 |
| | FMAB76 | 208 | 208 | 178 | 178 | 149 | 173 | 175 | 179 | 214 | 222 | 156 | 160 | 420 | 420 | 202 | 210 |
| | FMAB77 | 198 | 226 | 178 | 180 | 149 | 173 | 175 | 179 | 218 | 218 | 160 | 160 | 420 | 420 | 206 | 210 |
| | FMAB78 | 208 | 212 | 178 | 180 | 149 | 157 | 175 | 179 | 214 | 230 | 160 | 172 | 420 | 420 | 210 | 214 |
| | FMAB79 | 200 | 206 | 180 | 180 | 149 | 173 | 175 | 179 | 214 | 214 | 160 | 160 | 420 | 420 | 214 | 214 |
| | FMAB80 | 204 | 228 | 180 | 180 | 149 | 173 | 179 | 183 | 214 | 218 | 160 | 160 | 420 | 420 | 214 | 214 |
| | FMAB81 | 206 | 210 | 178 | 180 | 149 | 173 | 179 | 179 | 218 | 230 | 160 | 160 | 420 | 420 | 206 | 210 |
| Cabo Frio | FMRJ03 | ? | ? | 178 | 178 | 149 | 149 | 179 | 179 | ? | ? | 160 | 160 | 420 | 420 | 202 | 202 |
| | FMRJ04 | ? | ? | 178 | 180 | 149 | 149 | 179 | 179 | 214 | 226 | 160 | 160 | ? | ? | 230 | 230 |
| | FMRJ10 | 204 | 204 | 180 | 180 | 173 | 173 | 179 | 183 | 218 | 234 | 160 | 160 | 420 | 420 | 214 | 234 |
| | FMRJ11 | 204 | 204 | 178 | 180 | 145 | 149 | 171 | 179 | 214 | 214 | 160 | 160 | 428 | 428 | ? | ? |
| | FMRJ12 | ? | ? | 178 | 178 | 149 | 173 | 175 | 179 | 214 | 214 | 160 | 160 | ? | ? | ? | ? |
| | FMRJ13 | 206 | 236 | 178 | 180 | 149 | 149 | 179 | 179 | 214 | 218 | 156 | 160 | 420 | 420 | 214 | 238 |
| | FMRJ14 | ? | ? | 178 | 180 | 149 | 173 | 175 | 179 | 214 | 218 | 160 | 160 | ? | ? | ? | ? |
| | FMRJ15 | 204 | 206 | 178 | 180 | 149 | 173 | 179 | 179 | 214 | 214 | 160 | 160 | 424 | 432 | 214 | 218 |
| | FMRJ16 | ? | ? | 178 | 180 | 149 | 149 | 179 | 179 | 214 | 222 | 160 | 160 | 424 | 424 | 210 | 214 |
| | FMRJ69 | 204 | 206 | 180 | 180 | 149 | 173 | 179 | 179 | 218 | 222 | 160 | 160 | 420 | 420 | 214 | 234 |
| | FMRJ70 | 204 | 204 | 178 | 180 | 157 | 173 | 179 | 179 | 214 | 230 | 160 | 172 | 420 | 428 | 214 | 234 |
| | FMRJ71 | 204 | 204 | ? | ? | 149 | 173 | 179 | 179 | 214 | 222 | 160 | 160 | 424 | 424 | 210 | 214 |
| | FMRJ72 | 204 | 206 | 180 | 180 | 173 | 173 | 179 | 179 | 214 | 222 | 160 | 160 | 424 | 424 | 210 | 214 |
| | FMRJ73 | 204 | 204 | 182 | 182 | 149 | 149 | 179 | 179 | 214 | 218 | 160 | 160 | 420 | 428 | 234 | 238 |
| Cagarras | FMRC34 | 200 | 200 | 178 | 178 | 149 | 173 | 179 | 183 | 226 | 230 | 160 | 160 | ? | ? | 218 | 234 |
| | FMRC35 | ? | ? | 178 | 178 | 173 | 173 | 171 | 183 | 222 | 230 | 160 | 160 | 420 | 420 | 214 | 234 |
| | FMRC45 | 204 | 204 | 178 | 180 | 149 | 149 | 175 | 179 | 214 | 234 | 160 | 160 | 424 | 424 | ? | ? |
| | FMRC46 | 204 | 238 | 180 | 180 | 173 | 173 | 179 | 179 | 222 | 226 | 160 | 160 | 420 | 420 | 214 | 214 |
| | FMRC47 | 204 | 206 | 178 | 180 | 149 | 173 | 179 | 179 | 214 | 214 | 160 | 160 | 424 | 432 | 214 | 214 |
| | FMRC48 | 204 | 226 | 180 | 180 | 173 | 173 | 179 | 179 | 214 | 226 | 160 | 160 | 420 | 420 | 202 | 242 |

| | | | | | | | | | | | | | | | | | |
|---|---|---|---|---|---|---|---|---|---|---|---|---|---|---|---|---|---|
| | FMRC49 | 206 | 210 | ? | ? | 149 | 173 | 179 | 179 | 214 | 230 | 160 | 160 | ? | ? | ? | ? |
| | FMRC67 | 204 | 228 | 178 | 178 | 149 | 157 | 171 | 183 | 222 | 238 | 160 | 160 | 424 | 424 | 214 | 234 |
| | FMRC68 | 206 | 206 | 178 | 180 | 149 | 173 | 179 | 179 | 214 | 222 | 160 | 160 | 424 | 424 | 210 | 234 |
| Alcatrazes | FMAL82 | 206 | 240 | 178 | 180 | 149 | 149 | 175 | 179 | 214 | 222 | 160 | 160 | 420 | 420 | 210 | 210 |
| | FMAL83 | 204 | 206 | 180 | 180 | 149 | 149 | 175 | 179 | 222 | 230 | 160 | 160 | 424 | 424 | 214 | 214 |
| | FMAL84 | 204 | 204 | 180 | 180 | 173 | 173 | 179 | 179 | 214 | 234 | 160 | 160 | 424 | 424 | 210 | 214 |
| | FMAL85 | 204 | 204 | 178 | 180 | 157 | 173 | 175 | 179 | 214 | 226 | 160 | 160 | 420 | 428 | 214 | 234 |
| | FMAL86 | 204 | 204 | 178 | 180 | 149 | 173 | 175 | 179 | 214 | 230 | 160 | 160 | 420 | 428 | 234 | 238 |
| | FMAL87 | 204 | 204 | 180 | 180 | 173 | 173 | 175 | 179 | 230 | 230 | 160 | 172 | 420 | 428 | 218 | 230 |
| | FMAL88 | 204 | 226 | 178 | 180 | 173 | 173 | 175 | 179 | 214 | 222 | 160 | 160 | 428 | 428 | 210 | 210 |
| | FMAL89 | 200 | 206 | 178 | 180 | 149 | 173 | 171 | 183 | 222 | 222 | 160 | 160 | 428 | 428 | 210 | 238 |
| | FMAL90 | 206 | 228 | 178 | 180 | 149 | 173 | 175 | 179 | 214 | 222 | 160 | 160 | 420 | 420 | 210 | 214 |
| | FMAL92 | 200 | 200 | 178 | 178 | 149 | 183 | 175 | 179 | 222 | 230 | 160 | 160 | 420 | 428 | ? | ? |
| | FMAL93 | 200 | 200 | 178 | 180 | 175 | 175 | 179 | 179 | 226 | 230 | 160 | 160 | 424 | 424 | 214 | 234 |
| | FMAL94 | 204 | 204 | ? | ? | 149 | 149 | 179 | 179 | 214 | 214 | 160 | 160 | 424 | 432 | 206 | 234 |
| | FMAL95 | 202 | 236 | 180 | 180 | 149 | 173 | 179 | 179 | 214 | 218 | 160 | 160 | 424 | 424 | 210 | 214 |
| | FMAL96 | ? | ? | 178 | 180 | 157 | 173 | 179 | 183 | 222 | 234 | 160 | 160 | 420 | 420 | 210 | 214 |
| | FMAL97 | 206 | 228 | 180 | 180 | 173 | 173 | 179 | 179 | 214 | 214 | 160 | 160 | ? | ? | ? | ? |
| | FMAL98 | 204 | 204 | 180 | 180 | 157 | 173 | 175 | 179 | 226 | 230 | 160 | 160 | 420 | 420 | 214 | 234 |
| | FMAL 99 | 214 | 214 | 178 | 180 | 173 | 181 | 179 | 179 | 226 | 234 | 160 | 160 | 424 | 432 | 214 | 214 |
| | FMAL100 | 204 | 204 | 178 | 180 | 175 | 183 | 179 | 179 | 214 | 218 | 160 | 160 | 424 | 432 | 214 | 234 |
| | FMAL101 | 204 | 204 | 178 | 178 | 173 | 173 | 179 | 179 | 214 | 218 | 160 | 160 | 424 | 424 | ? | ? |
| Currais | FMC01 | 200 | 206 | 178 | 178 | 149 | 173 | 179 | 179 | 214 | 218 | 160 | 160 | 420 | 428 | 202 | 238 |
| | FMC02 | 204 | 232 | 180 | 180 | 175 | 175 | 179 | 179 | 214 | 214 | 160 | 160 | 424 | 424 | 210 | 214 |
| | FMC17 | 200 | 222 | 178 | 180 | 149 | 173 | 179 | 179 | 214 | 214 | 160 | 160 | 424 | 432 | 210 | 214 |
| | FMC18 | 222 | 232 | 180 | 180 | 149 | 173 | 179 | 179 | 214 | 214 | 160 | 160 | 424 | 424 | 210 | 210 |
| | FMC20 | 204 | 226 | 178 | 180 | 149 | 173 | 179 | 179 | 218 | 230 | 160 | 160 | 424 | 424 | 202 | 214 |
| | FMC32 | 204 | 220 | 180 | 180 | 173 | 173 | 179 | 183 | 214 | 222 | 160 | 160 | 420 | 420 | 214 | 218 |
| | FMC33 | 200 | 228 | 178 | 178 | 149 | 173 | 179 | 183 | 214 | 222 | 160 | 160 | 420 | 420 | 202 | 210 |
| | FMC42 | 204 | 204 | 180 | 180 | 149 | 149 | 175 | 179 | 226 | 238 | 160 | 160 | 420 | 420 | 210 | 234 |
| | FMC43 | 200 | 228 | 180 | 180 | 149 | 175 | 179 | 179 | 218 | 230 | 160 | 160 | ? | ? | 214 | 214 |
| Moleques do Sul | FMMS105 | 204 | 204 | 178 | 180 | 149 | 149 | 179 | 179 | 222 | 226 | 160 | 160 | 424 | 424 | 214 | 234 |
| | FMMS106 | 204 | 236 | 178 | 178 | 149 | 175 | 179 | 183 | 222 | 222 | 160 | 160 | 424 | 424 | 214 | 214 |
| | FMMS107 | 204 | 226 | 178 | 180 | 149 | 149 | 179 | 179 | 214 | 230 | 160 | 160 | 420 | 420 | 202 | 202 |
| | FMMS108 | 206 | 206 | 180 | 180 | 149 | 149 | 179 | 179 | 230 | 230 | 160 | 160 | ? | ? | 218 | 238 |
| | FMMS109 | 204 | 204 | 180 | 180 | 149 | 149 | 175 | 179 | 230 | 234 | 160 | 160 | ? | ? | 210 | 214 |

| | | | | | | | | | | | | | | | | |
|---|---|---|---|---|---|---|---|---|---|---|---|---|---|---|---|---|
| FMMS110 | 200 | 206 | 178 | 180 | 149 | 149 | 179 | 179 | 214 | 230 | 160 | 160 | 420 | 420 | ? | ? |
| FMMS111 | 200 | 204 | 186 | 186 | 149 | 149 | 179 | 179 | 222 | 230 | 160 | 160 | 424 | 424 | ? | ? |
| FMMS112 | 202 | 202 | 180 | 180 | 149 | 173 | 179 | 183 | 214 | 214 | 160 | 160 | 424 | 424 | 210 | 230 |
| FMMS113 | 204 | 204 | 180 | 180 | 149 | 173 | 175 | 175 | ? | ? | 160 | 160 | 424 | 424 | 214 | 230 |
| FMMS114 | ? | ? | 180 | 180 | 173 | 173 | 179 | 179 | 218 | 222 | 160 | 160 | ? | ? | 202 | 214 |
| FMMS115 | ? | ? | 178 | 180 | 149 | 173 | 179 | 183 | 218 | 230 | 160 | 160 | ? | ? | | |
| FMMS116 | 204 | 204 | 178 | 180 | 149 | 157 | 179 | 179 | 214 | 230 | 160 | 160 | 424 | 432 | 210 | 214 |
| FMMS117 | ? | ? | 178 | 180 | 149 | 173 | 179 | 179 | 214 | 226 | 160 | 160 | ? | ? | 214 | 238 |
| FMMS118 | 204 | 228 | 178 | 180 | 173 | 173 | 179 | 183 | 222 | 222 | 160 | 160 | ? | ? | ? | ? |
| FMMS119 | 200 | 200 | 180 | 180 | 149 | 149 | 179 | 179 | 214 | 214 | 160 | 172 | 420 | 420 | 214 | 226 |
| FMMS120 | 204 | 204 | 180 | 180 | 157 | 173 | 171 | 175 | 222 | 226 | 160 | 160 | 420 | 420 | 210 | 214 |
| FMMS121 | 204 | 204 | ? | ? | 173 | 173 | 179 | 179 | 218 | 230 | 160 | 160 | 424 | 432 | ? | ? |
| FMMS122 | 200 | 206 | 178 | 180 | 149 | 173 | 179 | 179 | 222 | 230 | 160 | 160 | 424 | 424 | 214 | 214 |
| FMMS123 | 200 | 206 | 178 | 178 | 149 | 173 | 179 | 179 | 214 | 226 | 160 | 160 | 424 | 424 | 214 | 214 |
| FMMS124 | 204 | 204 | 180 | 180 | 149 | 173 | 179 | 179 | 222 | 234 | 160 | 160 | 424 | 424 | 210 | 238 |
| FMMS125 | 204 | 206 | 178 | 178 | 173 | 173 | 179 | 179 | 222 | 226 | 160 | 160 | 424 | 424 | 214 | 234 |
| FMMS126 | 200 | 214 | 178 | 180 | 149 | 149 | 179 | 179 | 214 | 230 | 160 | 160 | 420 | 420 | 222 | 230 |

Notes: Missing data is represented by question marks “?”. Allele sizes are represented as the size in capillary electrophoresis.
