# Supplementary material for: Population Genetic Structure of the Magnificent Frigatebird Fregata magnificens (Aves, Suliformes) Breeding Colonies in the Western Atlantic Ocean
Source: PLoS One. 2016 Feb 22;11(2):e0149834. doi: 10.1371/journal.pone.0149834 (PMC4762693; doi:10.1371/journal.pone.0149834)
Supplement: S2 Table — (PDF) [file pone.0149834.s004.pdf]

S2 Table. Haplotype diversity and distribution in all sampled populations.

**Population**	**N**	**Hd**	**H1**	**H2**	**H3**	**H4**	**H5**
Barbuda	7	0.524 ± 0.209	0	1	5	1	0
Grand Connétable	14	0.670 ± 0.082	7	3	4	0	0
Abrolhos	7	0.286 ± 0.196	6	0	0	0	1
Cabo Frio	6	0.000	6	0	0	0	0
Cagarras	6	0.000	6	0	0	0	0
Alcatrazes	7	0.000	7	0	0	0	0
Currais	9	0.000	9	0	0	0	0
Moleques do Sul	8	0.000	8	0	0	0	0

N – sample size; Hd – Haplotype diversity ± standard deviation; H1 – H5 – mtDNA haplotypes (see text and Figure 2 for details).
