# Supplementary material for: Population Genetic Structure of the Magnificent Frigatebird Fregata magnificens (Aves, Suliformes) Breeding Colonies in the Western Atlantic Ocean
Source: PLoS One. 2016 Feb 22;11(2):e0149834. doi: 10.1371/journal.pone.0149834 (PMC4762693; doi:10.1371/journal.pone.0149834)
Supplement: S3 Table — (PDF) [file pone.0149834.s005.pdf]

S3 Table. Genetic diversity values for STR data excluding locus Fmin17.

**Population**	**N**	**N_A**	**AR**	**F_{IS}**	**H_O**	**H_E**
Barbuda	29	8.57	5.381	0.048	0.677 ± 0.222	0.710 ± 0.248
Grand Connétable	37	7.43	4.760	0.112	0.589 ± 0.206	0.662 ± 0.266
Abrolhos	18	5.57	4.129	-0.028	0.628 ± 0.223	0.611 ± 0.224
Cabo Frio	14	4.29	3.447	0.002	0.499 ± 0.242	0.501 ± 0.235
Cagarras	9	4.14	3.936	0.116	0.600 ± 0.186	0.577 ± 0.286
Alcatrazes	18	5.14	3.833	0.094	0.520 ± 0.246	0.571 ± 0.261
Currais	9	4.14	3.821	0.076	0.592 ± 0.259	0.547 ± 0.309
Moleques do Sul	22	5.14	3.724	0.155	0.457 ± 0.255	0.538 ± 0.267

N – sample size; N_A – Average number of alleles; AR – Allelic richness; H_O – Observed heterozygosity ± standard deviation; H_E – Expected heterozygosity ± standard deviation.
