# Supplementary material for: Population Genetic Structure of the Magnificent Frigatebird Fregata magnificens (Aves, Suliformes) Breeding Colonies in the Western Atlantic Ocean
Source: PLoS One. 2016 Feb 22;11(2):e0149834. doi: 10.1371/journal.pone.0149834 (PMC4762693; doi:10.1371/journal.pone.0149834)
Supplement: S4 Table — (PDF) [file pone.0149834.s006.pdf]

S4 Table. Pairwise F_{ST} (upper diagonal) and R_{ST} (lower diagonal) values for STR data excluding locus Fmin17.

**Population**	**1**	**2**	**3**	**4**	**5**	**6**	**7**	**8**
1- Abrolhos		<0.001	<0.001	<0.001	0.004	0.002	<0.001	<0.001
2- Cabo Frio	**0.086**		-0.007	-0.005	-0.004	-0.003	**0.005**	0.002
3- Cagarras	<0.001	<0.001		0.004	0.010	0.006	**0.005**	**0.007**
4- Alcatrazes	0.012	**0.069**	-0.020		0.004	0.001	<0.001	<0.001
5- Currais	-0.041	<0.001	-0.020	0.023		0.003	0.003	**0.004**
6- Moleques do Sul	**0.084**	-0.020	0.019	**0.073**	**0.066**		0.004	0.003
7- Barbuda	-0.004	**0.124**	0.021	**0.049**	-0.016	**0.147**		<0.001
8- Grand Connétable	-0.027	**0.062**	-0.013	0.012	-0.060	**0.088**	<0.001	

Values in bold are statistically significant (P<0.05).
