# Supplementary material for: Population Genetic Structure of the Magnificent Frigatebird Fregata magnificens (Aves, Suliformes) Breeding Colonies in the Western Atlantic Ocean
Source: PLoS One. 2016 Feb 22;11(2):e0149834. doi: 10.1371/journal.pone.0149834 (PMC4762693; doi:10.1371/journal.pone.0149834)
Supplement: S5 Table — (PDF) [file pone.0149834.s007.pdf]

S5 Table. Structure analysis for K=2 based on different assumptions. The values for each population represent the mean posterior probability across individuals of belonging to (or having ancestry on) each ancestry cluster estimated in the analysis.

Configuration Set	A		B		C		D		E		F	
Use of the Admixture model	No		No		Yes		Yes		Yes		Yes	
Use of LocPrior	No		Yes		No		Yes		No		Yes	
Use of loci Fmin17	Yes		Yes		Yes		Yes		No		No	
Ancestry cluster (K=2)	1	2	1	2	1	2	1	2	1	2	1	2
Barbuda	0.972	0.028	0.999	0.001	0.879	0.121	0.966	0.034	0.847	0.153	0.955	0.045
Grand Connétable	0.718	0.282	0.880	0.120	0.685	0.315	0.633	0.367	0.648	0.352	0.658	0.342
Abrolhos	0.288	0.712	0.033	0.967	0.338	0.662	0.199	0.801	0.389	0.611	0.288	0.712
Cabo Frio	0.027	0.973	0.001	0.999	0.162	0.838	0.023	0.977	0.151	0.849	0.019	0.981
Cagarras	0.063	0.937	0.001	0.999	0.226	0.774	0.060	0.940	0.253	0.747	0.093	0.907
Alcatrazes	0.125	0.875	0.002	0.998	0.271	0.729	0.060	0.940	0.247	0.753	0.041	0.959
Currais	0.029	0.971	0.001	0.999	0.158	0.842	0.031	0.969	0.202	0.798	0.043	0.957
Moleques do Sul	0.039	0.961	0.000	1.000	0.158	0.842	0.013	0.987	0.184	0.816	0.015	0.985
