# Supplementary material for: Population Genetic Structure of the Magnificent Frigatebird Fregata magnificens (Aves, Suliformes) Breeding Colonies in the Western Atlantic Ocean
Source: PLoS One. 2016 Feb 22;11(2):e0149834. doi: 10.1371/journal.pone.0149834 (PMC4762693; doi:10.1371/journal.pone.0149834)
Supplement: S6 Table — (PDF) [file pone.0149834.s008.pdf]

S6 Table. Migration estimates using BayesAss.

Population	1	2	3	4	5	6	7	8
1- Barbuda	0.8498 ± 0.0410	0.0638 ± 0.0373	0.0253 ± 0.0177	0.0093 ± 0.0090	0.0093 ± 0.0090	0.0096 ± 0.0096	0.0093 ± 0.0090	0.0236 ± 0.0179
2- Grand Connétable	0.0154 ± 0.0143	0.8322 ± 0.0360	**0.0939 ± 0.0330**	0.0076 ± 0.0074	0.0076 ± 0.0074	0.0079 ± 0.0080	0.0075 ± 0.0073	0.0279 ± 0.0190
3- Abrolhos	0.0130 ± 0.0124	0.0226 ± 0.0208	0.8713 ± 0.0397	0.0123 ± 0.0118	0.0122 ± 0.0117	0.0126 ± 0.0124	0.0124 ± 0.0119	0.0437 ± 0.0273
4- Cabo Frio	0.0152 ± 0.0144	0.0256 ± 0.0208	**0.0819 ± 0.0347**	0.6828 ± 0.0154	0.0152 ± 0.0144	0.0165 ± 0.0177	0.0152 ± 0.0145	**0.1476** ± **0.0387**
5- Cagarras	0.0193 ± 0.0183	0.0250 ± 0.0228	**0.0883 ± 0.0387**	0.0195 ± 0.0183	0.6879 ± 0.0197	0.0206 ± 0.0204	0.0196 ± 0.0184	**0.1199** ± **0.0401**
6- Alcatrazes	0.0125 ± 0.0120	0.0184 ± 0.0166	**0.1216 ± 0.0332**	0.0122 ± 0.0117	0.0122 ± 0.0118	0.6810 ± 0.0173	0.0121 ± 0.0116	**0.1300** ± **0.0353**
7- Currais	0.0196 ± 0.0184	0.0197 ± 0.0186	**0.1060 ± 0.0390**	0.0195 ± 0.0184	0.0196 ± 0.0185	0.0209 ± 0.0206	0.6877 ± 0.0196	**0.1070** ± **0.0399**
8- Moleques do Sul	0.0122 ± 0.0117	0.0126 ± 0.0121	0.0388 0.0234	0.0104 ± 0.0102	0.0104 ± 0.0101	0.0118 ± 0.0149	0.0104 ± 0.0102	0.8933 ± 0.0327

For each cell, m[i][j] is the fraction of individuals in population i that are migrants derived from population j (per generation). Values are presented as (estimate ± standard deviation). Values in bold are statistically significant considering an interval of 1.96xSD (see main text for details).
